# Understanding interactions between urban development policies and GHG emissions: A case study in Stockholm Region

**DOI:** 10.1007/s13280-019-01290-y

**Published:** 2019-11-20

**Authors:** Haozhi Pan, Jessica Page, Le Zhang, Cong Cong, Carla Ferreira, Elisie Jonsson, Helena Näsström, Georgia Destouni, Brian Deal, Zahra Kalantari

**Affiliations:** 1 School of Design, Shanghai Jiao Tong University, 800 Dongchuan Rd., Shanghai, 200240 China; 2 Department of Physical Geography and Bolin Centre for Climate Research, Stockholm University, 106 91 Stockholm, Sweden; 3 Department of Landscape Architecture, University of Illinois at Urbana-Champaign, Temple Buell Hall 611 Taft Drive, Champaign, 61820 IL USA; 4 Escola Superior Agrária de Coimbra, Bencanta, 3045 Coimbra, Portugal; 5 Regional Planning, Growth and Regional Planning Management, Stockholm, Sweden; 6 Department of Urban and Regional Planning, University of Illinois at Urbana-Champaign, Temple Buell Hall 611 Taft Drive, Champaign, 61820 IL USA

**Keywords:** Climate change, Greenhouse gas emissions, Land-use change, Planning support system, Social–ecological system, Stockholm

## Abstract

Human-induced urban growth and sprawl have implications for greenhouse gas (GHG) emissions that may not be included in conventional GHG accounting methods. Improved understanding of this issue requires use of interactive, spatial-explicit social–ecological systems modeling. This paper develops a comprehensive approach to modeling GHG emissions from urban developments, considering Stockholm County, Sweden as a case study. GHG projections to 2040 with a social–ecological system model yield overall greater emissions than simple extrapolations in official climate action planning. The most pronounced difference in emissions (39% higher) from energy use single-residence buildings resulting from urban sprawl. And this difference is not accounted for in the simple extrapolations. Scenario results indicate that a zoning policy, restricting urban development in certain areas, can mitigate 72% of the total emission effects of the model-projected urban sprawl. The study outcomes include a decision support interface for communicating results and policy implications with policymakers.

**Electronic supplementary material:**

The online version of this article (10.1007/s13280-019-01290-y) contains supplementary material, which is available to authorized users.

## Introduction

Understanding the role of human-driven land-use changes in climate change is important for our ability to select effective and efficient mitigation and adaptation strategies (Bierwagen et al. 2010). Interactive effects of climate and land-use changes affect social and ecological systems, and provision of ecosystem services by the latter (Destouni et al. 2013; Seung-Hwan et al. 2013; Pan et al. 2019a). Urbanization is an essential part of human-driven land-use change, and its impacts need to be considered in regional climate modeling (Hu et al. 2015). For example, Wilson and Weng (2011) show important soil and water implications for the Midwestern US when accounting for urbanized land use (impervious surfaces) in an integrated downscaled climate model.

Greenhouse gas (GHG) emissions and carbon sinks associated with urbanization are relatively well studied (Lubowski et al. 2006; Searchinger et al. 2008; Larsen and Hertwich 2010; Han et al. 2017). For example, Han et al. (2017) found significant increases in carbon emission sources and simultaneous loss of carbon sinks associated with fast urbanization in the Yangtze River Delta, China. Lubowski et al. (2006) model land uses in the contiguous US and suggest that future climate strategies need to consider forest-based carbon sequestration. Searchinger et al. (2008) use a worldwide agricultural model to estimate emissions from land-use change and show that cropland conversion for biofuels nearly doubles greenhouse emissions over 30 years in California. Larsen and Hertwich (2010) calculate the carbon footprint for 429 Norwegian municipalities and show that it changes significantly depending on municipality size and wealth level. Urban planning and policy tools are needed to identify and efficiently mitigate land-use changes associated with urbanization that pose major climate and environmental risks (Hobbs et al. 2016; Deal et al. 2017a; Pan et al. 2018b). Hobbs et al. (2016) present a regionally calibrated model for South Australia that collects new information to facilitate better decisions in regional land-use planning for reforestation and carbon sequestration. Recent progress in methods of emission accounting and policy analysis includes climate and ecosystem scenarios with implications for future urban land-use patterns (Pan et al. 2018a). Spatial-explicit assessments of the climate impacts and feedbacks of urbanization and associated land-use changes can provide substantial policy support in selecting priority areas for emission reductions (Pielke et al. 2002).

Models that capture interactive effects and feedbacks between climate and land-use/cover changes at high spatial resolution (finer than 100-m resolution) and over a mid- to long-term (such as future 30 years) temporal horizon can improve representation and capture of climate impacts on human social systems and human reactions (Bierwagen et al. 2010; Pan et al. 2019b). This can improve understanding and projection of future outcomes and impacts and inform strategies for GHG emission mitigation and adaptation (Pan et al. 2018b). Moreover, urban growth is often associated with increased energy use and associated GHG emissions (Chau et al. 2015; Kraucunas et al. 2015; Nejat et al. 2015; Gren et al. 2019). Change in urban form, through land-use change, also has transportation implications and can significantly influence travel demand (Hankey and Marshall 2010). Comprehensive social–ecological assessment of urban land uses and GHG emissions thus needs to incorporate and couple multiple economic, land use, transportation, climate, and other environmental factors (Bercht and Wijermans 2019; Carrière 2019; Ehrich et al. 2019; Valencia et al. 2019).

Besides increasing energy use and associated GHG emissions, urbanization is likely to simultaneously reduce carbon sinks, thus exacerbating climate change impacts. Climate change can in turn affect urban development decisions. For example, increased (or decreased) rainfall or flood risk can affect the expected economic efficiency of land development, thereby limiting the location and configuration of new urban developments (Deng et al. 2013). Deng et al. (2013) explore such interactions through regional climate modeling examining how social activities may be exposed to extreme climate events. Overall, climate and land-use modeling need to account for multiple social and ecological system interactions and feedbacks (such as climate impacts on human socio-economic activities, and human reactions that change emission patterns and volumes), in order to provide accurate and comprehensive scenario analysis to support relevant policy decisions.

In this study, we have developed and applied a process-based coupled social–ecological modeling approach to identify the complex interactions, and their effects and feedbacks, between urbanization and associated land-use changes and climate change. The model builds on existing approaches for constructing interactive and policy-driven scenarios of changes in (i) land use and (ii) GHG emissions. The modeling approach is applied to Stockholm County, Sweden. This was selected as case study since the region has high demand for urban growth due to projected increases of over 30% in population and employment from 2014 to 2040 (Tillväxt och regionplaneförvaltningen, TRF 2017). Such growth can induce high levels of associated GHG emissions. The major city in the region, Stockholm, the Swedish capital, is also a pioneer in climate action planning and has the planning capacity to use policy instruments to mitigate future emissions (Stockholm City 2016). The case study addresses the following main research questions: What are the aggregated climate impacts (focusing on GHG emissions and carbon sink losses) of urban land-use changes associated with building and transportation developments? Can improved ability to identify high-emission areas affect the design of climate change mitigation strategies, by appropriately restricting land development to achieve GHG emission goals?

Modeling of social–ecological processes and system feedbacks is necessary to answer these questions and test policy alternatives. Such modeling can also lead to further research developments and advances in knowledge of socio-economic interactions with climate and environmental changes through land-use changes. The methodology developed and applied in this study can be used for re-evaluation of baseline emission scenarios, based on more realistic accounting of urban land-use effects and feedbacks.

## Materials and Methods

### Study area and data

**Fig. 1:**
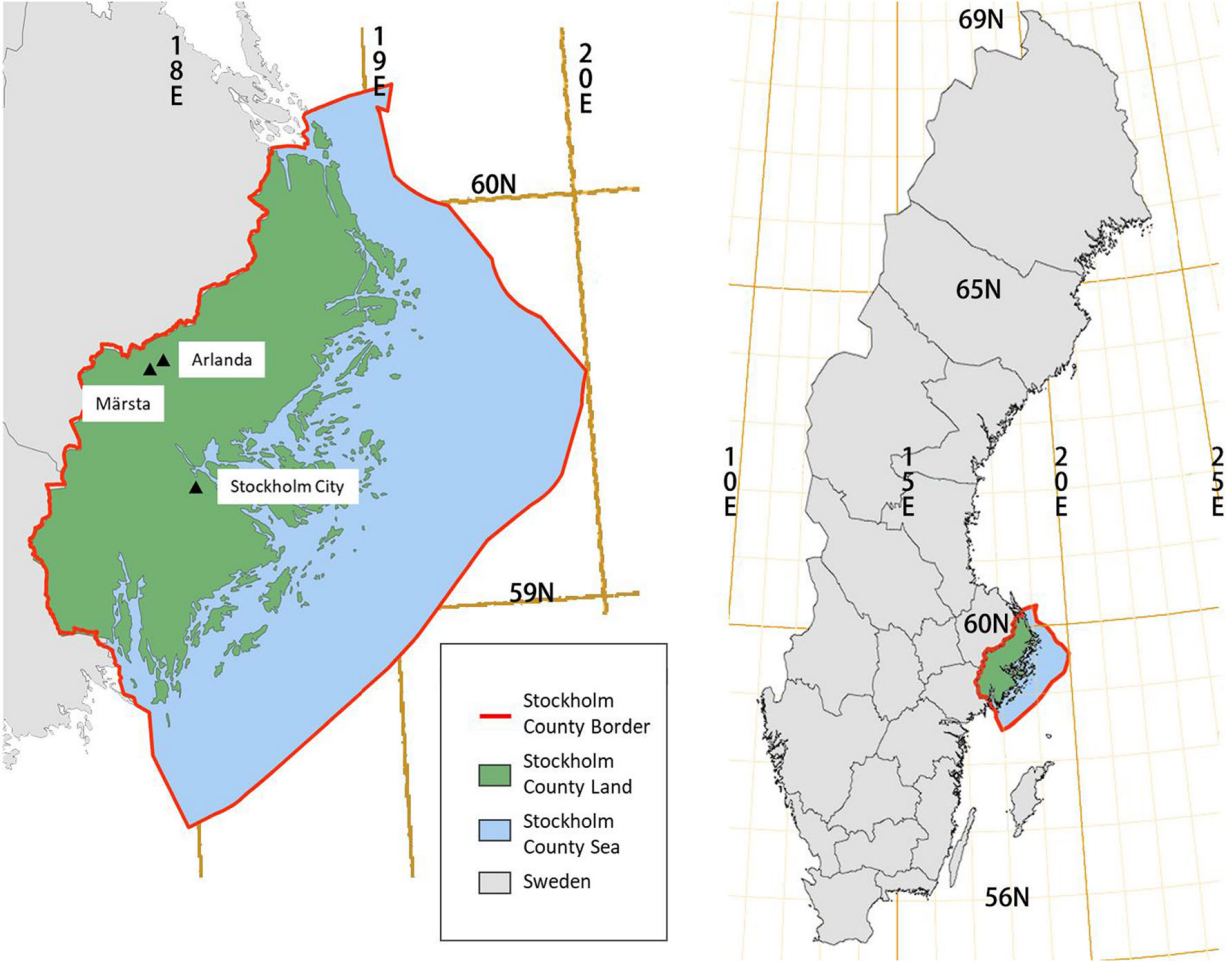
Stockholm County (green area, left map), located in the mid-eastern region (green area, right map) of Sweden

Stockholm County is the largest metropolitan region in Sweden. It includes the Swedish capital Stockholm, and the Stockholm Archipelago, which extends out into the Baltic Sea (Fig. 1). Stockholm County is located in the boreo-nemoral mixed-forest biome (Elmhagen et al. 2015) and its landscape includes urban areas (approximately 35% of total regional area), urban green spaces (7%), open water (both lakes and sea, 23%), coniferous (24%) and mixed (coniferous/deciduous, 4%) forests, and arable land (7%) (Goldenberg et al. 2017).

The Stockholm region has recently experienced major population growth. In 2018, 2 315 612 people lived in Stockholm County, representing 22.3% of Sweden’s total population (TRF 2017). According to the regional planning office, TRF, the population of Stockholm County is expected to grow by nearly 30% from 2014 to 2040, to around 2 800 000 inhabitants (TRF 2017). The region aims to be a worldwide leader in reducing GHG emissions, with the City of Stockholm having committed to being fossil fuel free by 2050 (a deadline recently brought forward to 2040), based on its *Strategy for a fossil*-*fuel free Stockholm* (*Strategy 2040*) (Stockholm City 2016).

The emission quantifications from *Strategy 2040* are used and adapted for the modeling scope of this paper. Geographically, we model Stockholm County, which is a larger area than Stockholm City and its vicinity (as the focus of *Strategy 2040*), in order to also capture GHG emissions from the incoming-outgoing transportation of the main urban area in the region. The transportation emissions in the larger geography are obtained from Stockholm County’s climate planning document (TRF 2018). For the emission scope of this paper, we also model land-use development beyond Stockholm City in order to estimate future emission growth in energy use of buildings (not considering the building manufacturing activities). Furthermore, this study accounts only for road and rail passenger vehicles in the transportation emissions. Adapted from *Strategy 2040*, we thus consider total emissions for Stockholm City of 3 460 000 tons of carbon dioxide equivalents (CO_2_e) for year 2014. Energy use in buildings contributes 1 600 000 tons and road and rail passenger travels contribute 1 860 000 tons of CO_2_e to these total emissions.

Beyond Stockholm City that, with roughly 1 million inhabitants, is the core urban area of Stockholm County, the present analysis also focuses on three northern suburban municipalities that are projected to host most of the future urban growth demand (TRF 2017). These are the Upplands-Bro Municipality (major growth in the Brunna area), with population of around 25 000 in 2015 and a substantial proportion of agricultural land; the Vallentuna Municipality (major growth in the Brottby area), with population of around 30 000 in 2015 and many single-family houses in high-quality natural amenities; and the Sigtuna Municipality (main growth associated with businesses relating to Arlanda International Airport in Märsta), with around 45 000 inhabitants in 2015; the data and statistics for these municipalities are obtained from Statistics Sweden (2018).

Data for the overall Stockholm County case include existing features of the region, in a digital terrain model (DEM) with 30 m × 30 m resolution, and existing population and job locations, land uses, roads, and public transport networks. These data have been provided by TRF, the Regional Development and Planning Department at Stockholm County Council. Land-use data are compiled from Urban Atlas (SE001L1_STOCKHOLM _UA2012) data and updated by Corine Land Cover (CLC European seamless vector database version 18_5) data to the land use of 2014. Data on planned future developments are obtained from the Regional Development Plan for the Stockholm County for 2050 (*Regional Utvecklingsplan För Stockholmsregionen*, RUFS 2050, TRF 2017). The future development data include planned road and public transport developments, “no-growth” zones (such as protected natural areas where no development is allowed), development areas, types of developments, development priorities, and demographic projections for the period 2015–2040. Base year data for 2014 from *Strategy 2040*, such as GHG emissions and total energy usage, are used as inputs in modeling.

### Framework for social–ecological process modeling and integration in policy decision support

**Fig. 2:**
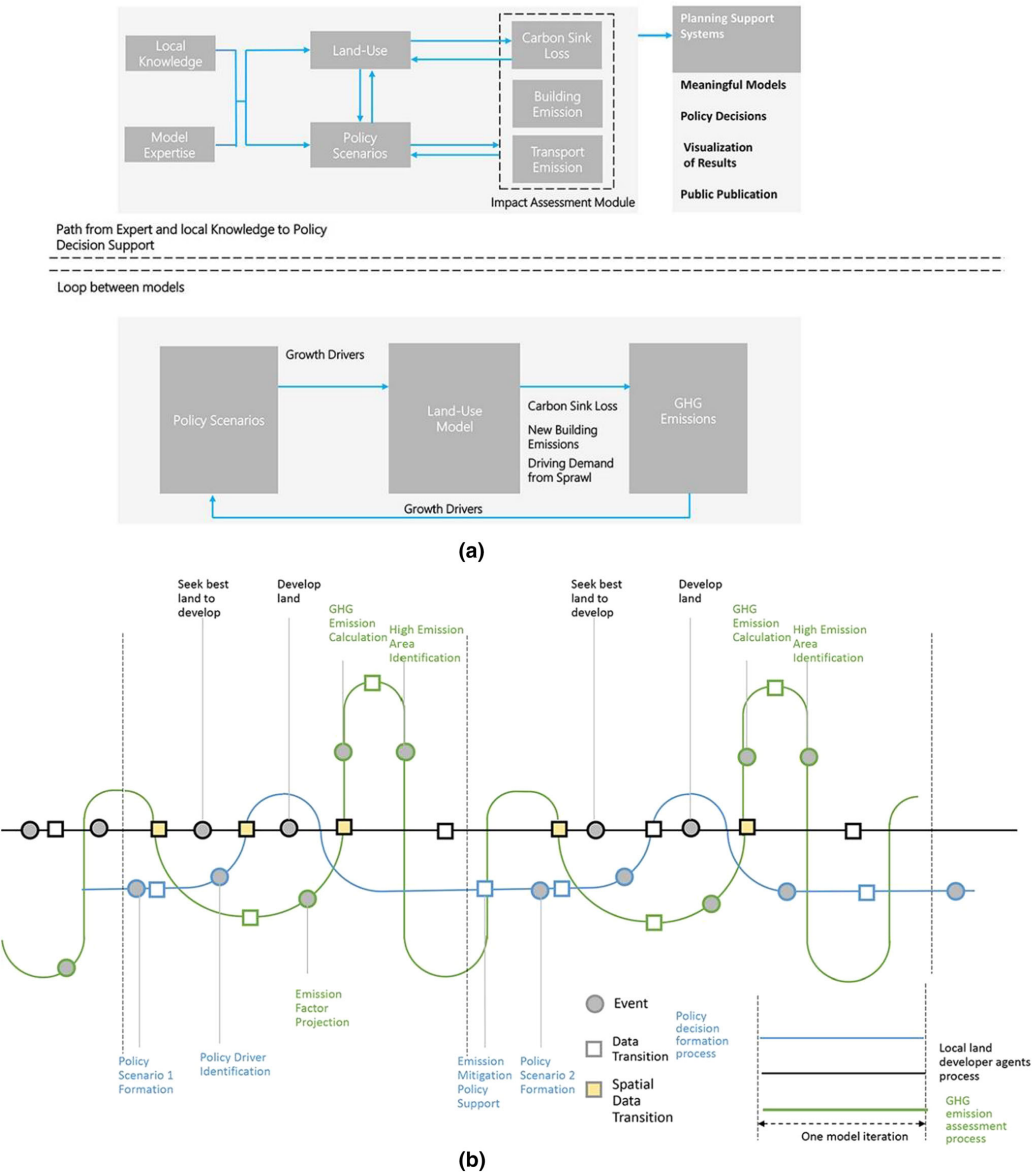
**a** Framework for social–ecological process and system modeling and pathway to policy decision support and **b** processes and feedbacks of coupled policy scenario, land use, and GHG emission assessment. Event means environmental changes (such as change of total carbon sink values from land-use change) or human actions in the social–ecological model (such as policy reactions to emissions surpassing certain amount)

An integrated modeling framework is proposed and used here to investigate the regional GHG emissions within a dynamic urbanization context, and thus assess the impact of policy scenarios. The framework is the result of a multidisciplinary collaboration between scientists and stakeholders. It includes (1) complex models for land use, GHG emissions, and policy scenarios, and (2) a pathway for transferring multidisciplinary modeling expertise into useful policy practices, through comprehensive modeling accounting for feedbacks (Fig. 2a).

In the coupled system model, land-use scenarios are not static assumptions, but are based on a dynamic complex urban systems model that links socio-economic policy scenarios, land-use decisions, and associated GHG emission impacts. The physical availability of land for commercial and residential developments is forecast via bottom-up land-use change probabilities. This modeling approach requires integration and justification of both human (socio-economic and land-use policies) and ecological (GHG emissions associated with human activities that have global climate impacts) processes. The coupled model provides a process-based understanding of GHG emissions associated with urbanization and human-driven land-use changes, through assessment of carbon sink losses, emissions from new residential and commercial buildings, and transportation emissions associated with urban sprawl. It allows the environmental impact of various policies to be tested, and therefore leads to more informed decisions. The iterative and interactive modeling process is illustrated in Fig. 2b.

### Land-use and impact modeling

#### Land-use change model for complex urban systems

In the proposed framework, as in other recent relevant modeling approaches for the Stockholm region (Kalantari et al. 2014, 2019; Pan et al. 2018b), the Land-Use Evolution and Impact Assessment Model (LEAM) is used to forecast scenarios of land-use changes in the study region. In LEAM, the land-use transformation potential of individual cells is evaluated by explicitly quantifying the forces (drivers) that contribute to change. Knowledge and account of the causal mechanisms of change provides local decision makers with the opportunity to test policy and investment choices and is a critical component for completing scenario planning exercises. Driver sub-models are locally dependent and derived through both analysis and local stakeholder interaction. An open architecture and modular design facilitates incorporation of additional local drivers, which are needed to improve the explanatory power of the model. A connectivity-based approach is used to measure city and employment centers for current land-use cells, and the outcome is used to identify highly probable re-development areas among existing developed land. Probability maps of future commercial and residential growth are built based on calibration. Future land-use allocations are subject to probability maps and population/employment growth projection based on scenarios identified. Details of LEAM calibration, validation, and variable specifications are documented in Supplementary Materials (S1).

#### Carbon sink assessment

In order to assess how future urban development and land-use change affect carbon sinks in Stockholm, driven by policy scenarios, social–ecological processes need to be coupled. This allows land cover conversions to be located from the urban growth process. It also allows associated carbon sink losses to be calculated based on the number of land cover cells (30 m × 30 m scale) originally representing high-value carbon sinks (such as wetlands and forests with trees of young ages) that are converted into urban built-up areas (residential or commercial cells). We have created a carbon sink map of the study region based on the latest available land-use map (TRF 2017), considering six classes: forest, shrubs, grass, cultivated crops, pasture, and wetlands (including both woody wetlands and herbaceous wetlands). Within forest areas, carbon sink values in the model are assigned considering different vegetation types and ages, given the carbon sequestration potential of (i) young and productive forests, and (ii) established or naturally occurring forests. Forest age and type data are obtained from Copernicus Forests Dominant Leaf Type (DLT-2015-20m). Details of carbon sink mapping are provided in Supplementary Materials S1.

#### Assessment of emissions from residential and commercial buildings

One of the major sources of carbon emissions associated with urbanization is energy use in buildings and associated commercial activities. In most current climate planning practices (including *Strategy 2040*), demand for new building construction is extrapolated from official population and employment forecasts. The main variable determining building energy use is building standards, including their requirements for technological improvements (such as better insulation, more efficient heating and cooling system, occupancy control systems). One key socio-economic and spatial-related factor that has so far been overlooked in residential GHG emission forecasts of existing climate action plans is the consequence of low-density residential developments associated with urban sprawl. Song and Knaap (2004) and Irwin and Bockstael (2007) report an increased tendency to build single-family houses with urban sprawl, resulting in higher GHG emissions per capita. Spatial-explicit land-use simulations can pinpoint new residential and commercial developments for regions with historic density and development pattern information. For example, simulated new urban growth that occurs at the urban fringe or suburban areas is more likely to involve single-family houses and large manufacturing companies (Pan et al. 2019b). On the other hand, in-fill developments at the urban core are more likely to involve high-density apartments and offices. Integrating land-use simulation and local density information can improve estimation of future building energy use. Details of the methods used for building energy emission estimation are provided in Supplementary Materials S1.

#### Assessment of transportation emissions

Previous studies have found that vehicle kilometers traveled (VKT) in and through an urban area increase with population and employment growth, and that total driving demand is also closely related to the urban form (Ewing et al. 2008; Cervero and Murakami 2010). Low-density and sprawl development can drive and increase GHG emissions (Liu and Shen 2011).

In this study, associated transportation emissions for 2040 are calculated based on the LEAM forecast of urban expansion (including urban form), together with passenger-vehicle GHG emissions calculated using the linear population density function of Hankey and Marshall (2010). Detailed information is provided in Supplementary Materials S1.

One important advantage of spatial-explicit modeling of land-use development is that change in future urban form can be forecast, along with total growth. As a result, VKT can be better estimated based on the new urban form forecast. The impacts of transportation systems on future urban development can be more accurately predicted by combining VKT estimates and projections on vehicle and fuel technology.

### Policy scenario analysis

As key human feedbacks in social–ecological systems, the model simulates local government reaction to potential GHG emissions as a LEAM “mitigation zoning” (MZ) scenario, comparing its emission outcomes to those of the *Strategy 2040* baseline scenario, and the LEAM reference scenario. The key assumptions in each scenario are listed in Table 1 and described below. Note that all scenarios use the same population change (from 2 163 000 in 2014 to 2 800 000 in 2040) and employment growth projection (from 1 150 000 in 2014 to 1 500 000 in 2040). The difference between the scenarios is the urban growth patterns with the new growth and effects on GHG emissions.

**Table 1:** Key scenario assumptions

Scenarios	Key assumptions
Baseline (*Strategy 2040*)	The 2040 GHG emissions is projected based on the proportion projected population increase based on *Strategy 2040* (Stockholm City 2016)
Reference (LEAM)	The 2040 GHG emissions is simulated based on a spatial-explicit social–ecological model with how new commercial and residential development to host the growing population would have implications on carbon sink, building, and transportation energy use
Mitigation zoning (LEAM)	The 2040 GHG emissions is simulated based on the reference (LEAM) model, while residential and commercial growth that could lead to high future GHG emissions are designated as no-growth zones

To address feedbacks of human reaction to human-driven climate change, the social–ecological modeling approach uses dynamic (annual) information on GHG emissions to investigate the LEAM MZ policy scenario, as an example of mitigation/adaptation response measures in future urban developments. The key assumption in the MZ scenario is that policymakers have strong awareness of urban growth and land-use change interactions, and their associated generation of GHG emissions (spatial emission intensity), and make adaptive policy changes at 5-year intervals.

The policy instrument considered in the model is the designation of special zones with restricted residential and commercial developments. Several types of special zones are identified and simulated by LEAM. The first type is future flood zones under expected climate change scenarios. This simulates policymakers’ awareness of climate change impacts prompting them to actively adapt future development strategy for climate mitigation. In the second type, 2040 flooding zones are projected by a hydrological model (r.sim.water) and a hydrodynamic model (MIKE FLOOD) with LEAM land-use change inputs. This simulates policymakers’ awareness of the necessity for climate adaptation. The third type is referred to as other no-growth zones, such as forest preserves, parks, and water bodies. Areas associated with high emission potential (such as large patches of forests occupied by urban development, regions far from urban cores, or single-family residence areas), flooding potential, or other types of restrictions are set as no-growth zones. As a result, residential and commercial developments are shifted to places with lower emission and flooding potential, although socio-economic attractiveness (measured by the LEAM probability map) may be slightly lower in those places.

Current GHG emission planning usually adopts energy (such as renewable energy), vehicle and building technology, or behavior-related policy to reduce future carbon emissions. In this study, we consider innovative spatial zoning, based on feedback from spatial-explicit model results, as an additional policy instrument available for policy makers to mitigate GHG emissions, with an assumed 5-year planning cycle for policy implementation and adjustment. GHG emissions from this spatial zoning policy scenario are compared with those in the *Strategy 2040* baseline and the LEAM reference scenarios. Full details of the MZ policy scenario and feedback modeling are provided in Supplementary Materials S1.

## Results

### Projected land-use change and urban expansion

**Fig. 3:**
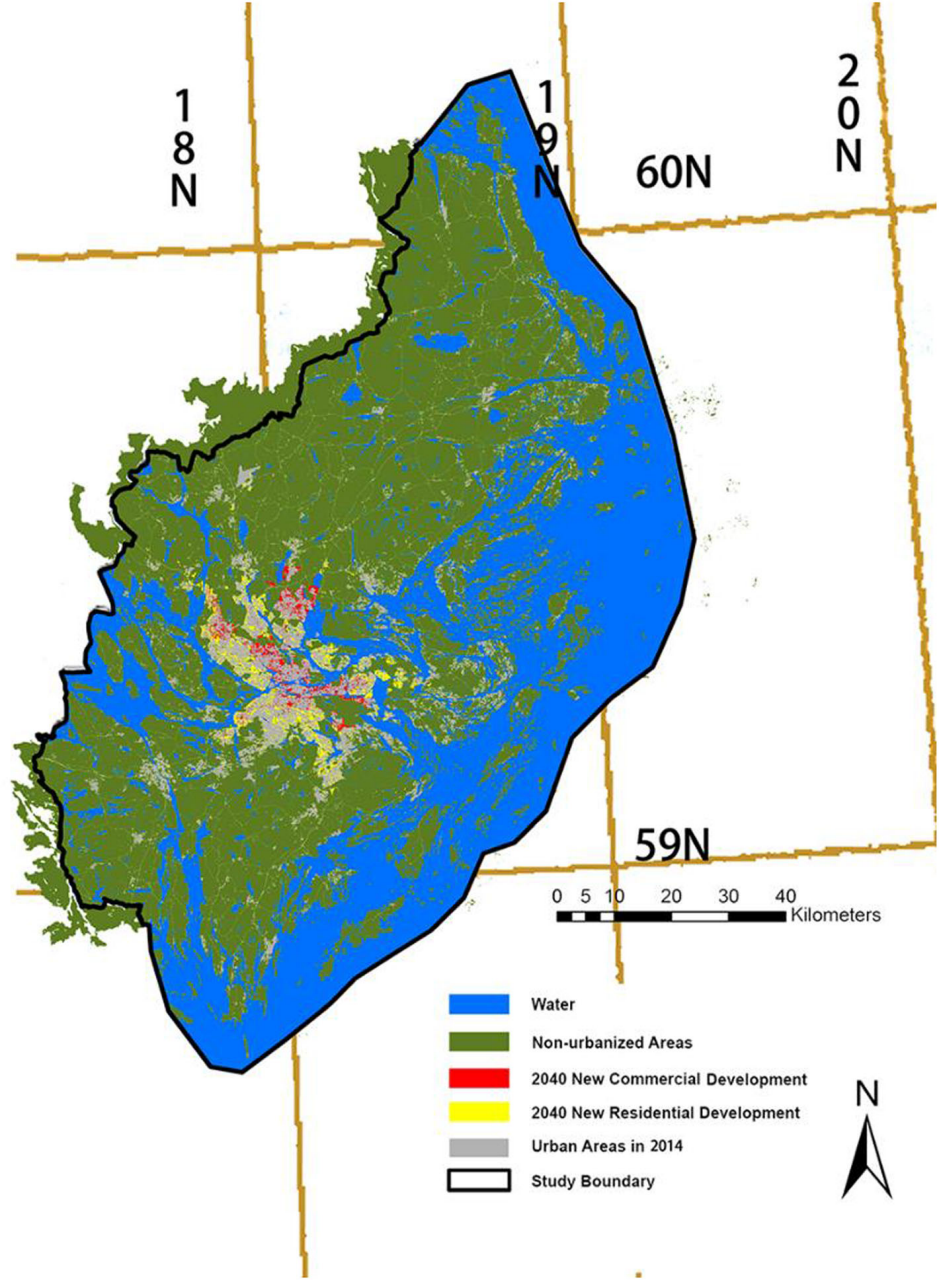
Land-use change projection in the LEAM reference scenario for the 2014–2040 period in the Stockholm region

The projected urban expansion is shown in Fig. 3. An area of 1.40 km^2^ of single-family homes and 10.82 km^2^ of multi-family homes can be expected to be built in Stockholm region by 2040. According to *Strategy 2040*, current residents live in about 34 km^2^ of total residential building stocks of different types. Total new development (12.22 km^2^) is expected to host about 30% of the new population (about 600 000) in Stockholm. Thus, the simulated residential developments have on average similar number of residents per area as the current housing stock. Most of the new development is expected to occur in the existing urban center, with some developments also occurring in sub-centers, including the Märsta and Arlanda Airport regions. The results show that Stockholm has a lower tendency for sprawl development than many other cities around the world, especially in North America (Pan et al. 2019b). Nevertheless, the rural developments in Märsta and other northern suburbs may be associated with higher than expected residential and transportation energy use.

### Projected carbon sinks

In the LEAM reference scenario, the changes from current to future land-use developments lead to carbon sink loss at a peak annual rate of 20.3 KT CO_2_e in 2040. This is equivalent to loss of 2.4% of the 2014 total carbon storage potential (CO_2_e/year) in the Stockholm region. Aggregating the carbon sink losses to their total cumulative sink value until 2040 (if they were not removed in the land-use developments) leads to a decrease in storage potential of around 2.3 MT CO_2_e.

### Projected GHG emissions

In the LEAM reference scenario, the projected new buildings yield a total increase in residential energy use of 1.27 GWh/m^2^. Based on a similar assumption as in *Strategy 2040* (31% emission reduction in building energy use for new developments compared with current buildings), total annual GHG emissions of 2.83 MT CO_2_e are expected by 2040 in the LEAM reference scenario, which is 39.4% more than in the *Strategy 2040* baseline scenario. Hence, urban sprawl with more single-family developments makes an essential difference and must be accounted for in projections of building-related GHG emissions.

In 2011, total passenger VKT of cars and public transit in Stockholm County was around 9.82 billion km/year, generating GHG emissions of 0.53 MT CO_2_e. In the LEAM reference scenario, the projected VKT for 2040 is 13.55 billion km/year, implying emissions of 2.63 MT CO_2_e/year, if public network expansion is assumed to develop at the same rate as the VKT growth. Thus passenger-vehicle emissions may almost double if no public transportation or fuel economy measures are taken to mitigate the growth in travel demand. This estimate of the LEAM reference scenario implies (13.8%) lower transportation emissions than in the baseline scenario of *Strategy 2040* (3.05 MT CO_2_e/year). However, it is then important that public transportation networks are expanded in line with the overall increasing transportation needs, in order to mitigate the growth in transportation emissions associated with the urban land-use growth.

In total, the LEAM reference scenario emissions from both energy use in buildings and associated transportation, in combination with the carbon sink losses, are 5.48 MT CO_2_e/year. These are 7.9% higher than the extrapolated emissions of 5.08 MT CO_2_e/year in the *Strategy 2040* baseline scenario. Even though this total difference is relatively small, it is important that it indicates the simple extrapolation as non-conservative, i.e., tending to underestimate rather than overestimate future total emissions.

### Mitigation zoning policy scenario

**Fig. 4:**
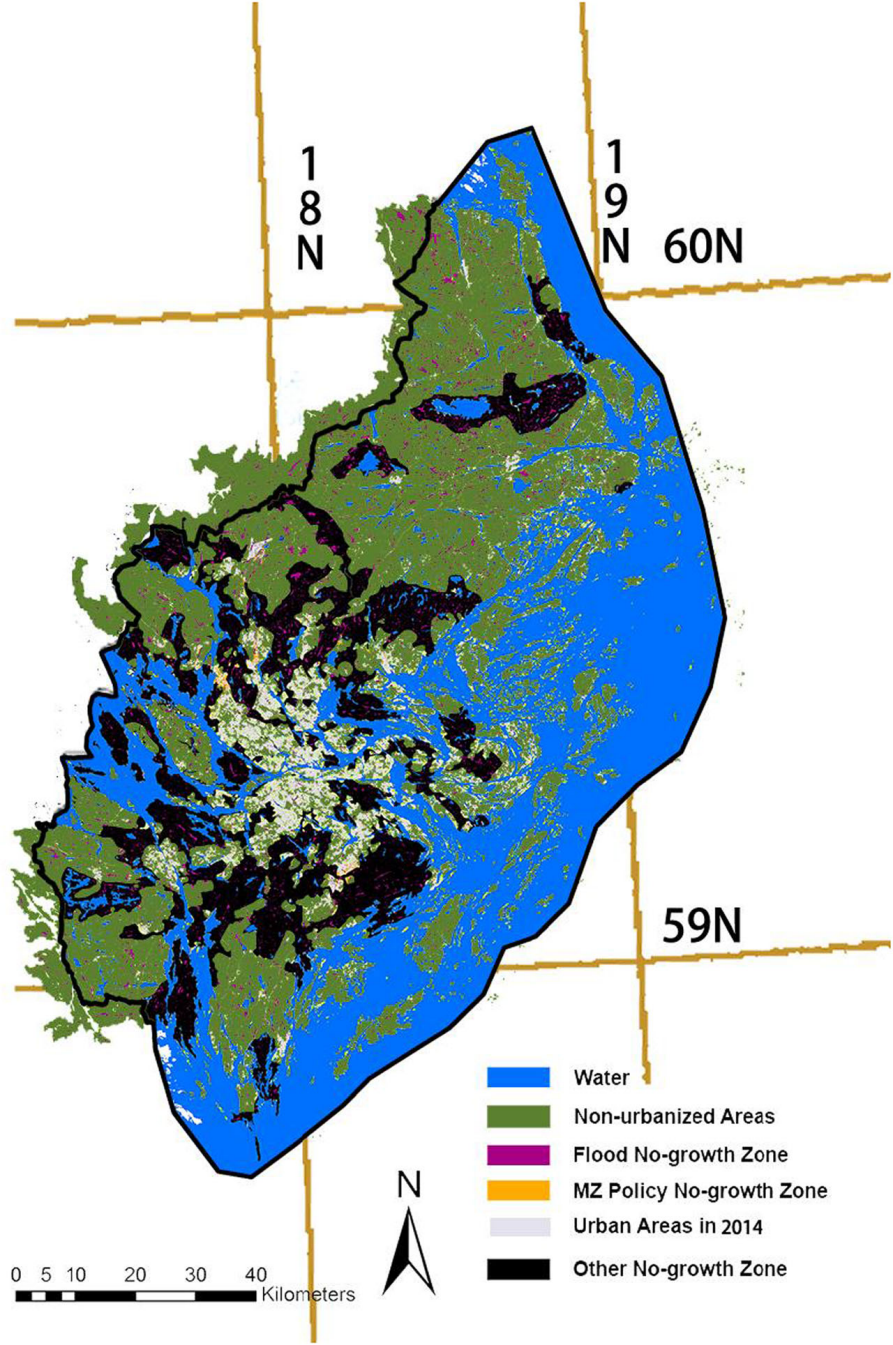
No-growth areas in the LEAM mitigation zoning (MZ) scenario for the Stockholm region by 2040

The no-growth zones with restricted residential and commercial developments in the LEAM MZ scenario for 2040 are shown in Fig. 4.

**Fig. 5:**
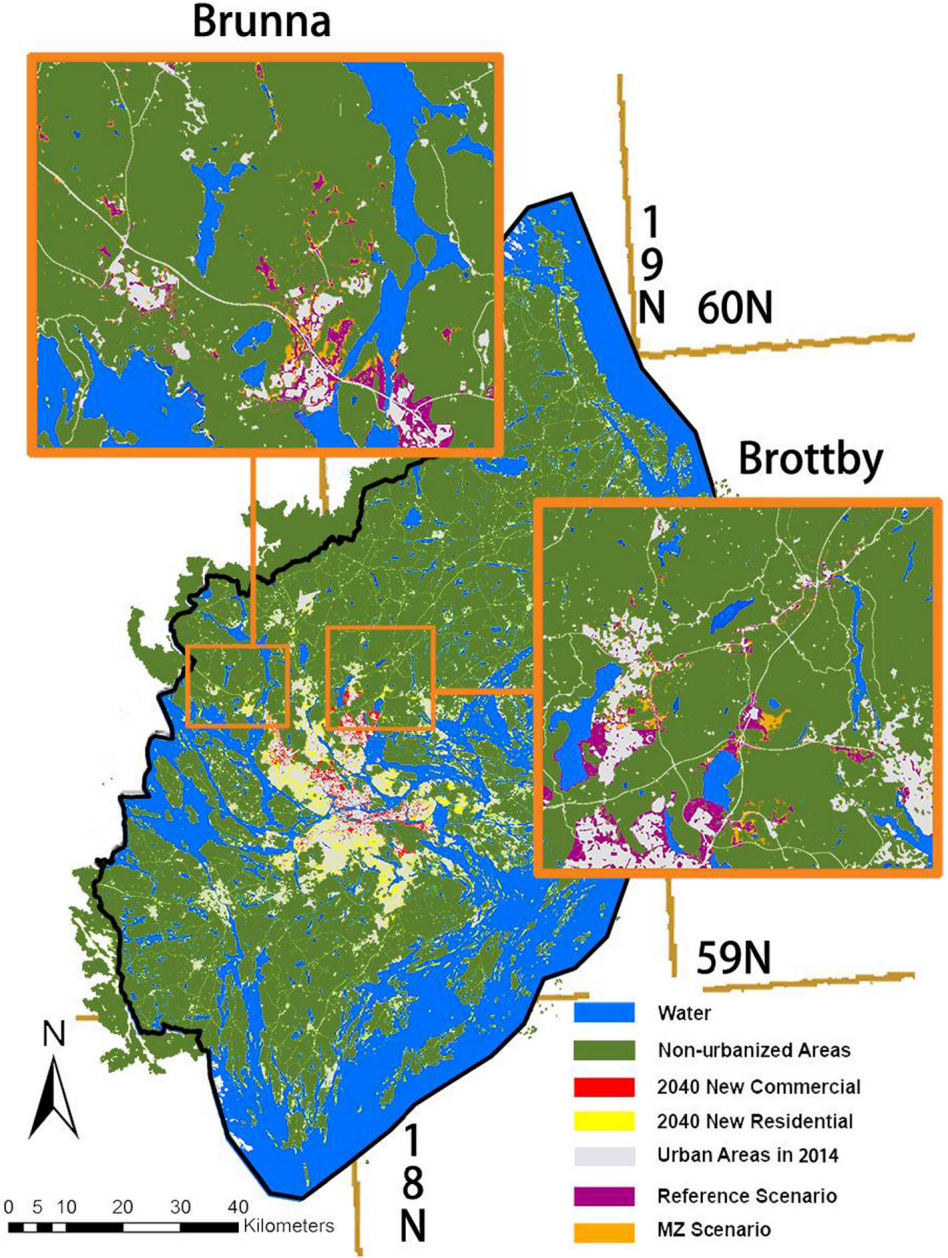
Location of urban developments in the LEAM mitigation zoning (MZ) scenario for the Stockholm region by 2040. The Brunna and east Brottby urban clusters are highlighted because urban developments are largely shifted there in the MZ scenario

Figure 5 further shows the new development pattern in the LEAM MZ scenario, highlighting areas to which developments are relocated in comparison with the LEAM reference scenario. Two types of developments in the reference scenario do not occur in the MZ scenario (Fig. 5): (i) urban developments in forest areas in the central city fringe, due to high carbon sink potential there, and (ii) urban developments in the Märsta and Arlanda Airport regions, which are limited due to their high emission potential as they generate major demand for travel/transportation to other developed areas in the city. Development in the MZ scenario is instead shifted to the Brunna and east Brottby urban clusters, which have relatively low carbon sink potential and require only about 70% of the travel time and distance to the main urban core compared with the Märsta and Arlanda Airport regions.

Comparison of projected carbon sink losses and GHG emissions in the LEAM reference and MZ scenarios (Table 2) shows that in the MZ scenario: (i) carbon sink losses are reduced by 64.5%, to 7.20 KT CO_2_e/year (from 0.02 to 0.01 MT CO_2_e/year if rounded as in Table 2), (ii) transportation emissions are reduced by 6.7%, to 2.72 MT CO_2_e/year, and (iii) building emissions are reduced by 3.7%, to 2.46 MT CO_2_e/year. This leads to a 5.3% reduction in total GHG emissions (including carbon sink losses), to 5.19 MT CO_2_e/year in the LEAM MZ scenario compared to the 5.48 MT CO_2_e/year in the LEAM reference scenario without a MZ policy. This emission decrease by 0.29 MT CO_2_e achieved by the MZ policy instrument corresponds to around 72.5% of the GHG emission increase caused by the urban growth and land-use changes (i.e., of the 0.4 MT CO_2_e difference between the 5.48 MT CO_2_e emissions of the LEAM reference scenario and the 5.08 MT CO_2_e emissions assumed in the baseline scenario of *Strategy 2014*). The LEAM MZ scenario still has higher total GHG emissions than the *Strategy 2040* baseline scenario, because the urban growth-induced increases in GHG emissions cannot be addressed by urban growth management policies alone. Additional measures, such as technology improvements, building standards, and transportation policies, are needed to complement urban growth management in addressing the associated emission increases.

**Table 2:** GHG emissions driven by carbon sink losses and new developments (building and transportation network) in (i) the existing condition (2014), when the *Strategy 2040* plans are published, (ii) the “Strategy 2040 Baseline,” based on current land-use trends, (iii) the “Reference Scenario,” which integrates both socio-ecological process (LEAM) and GHG emission models; and (iv) the “Mitigation Zoning Scenario,” assuming political strategies for spatial restrictions to new urban developments in 2040

Scenarios	Carbon sink loss (MT CO_2_e/year)	GHG emissions (MT CO_2_e/year)		Total (MT CO_2_e/year)
		Building (excluding manufacturing activities)	Road passenger-vehicle transportation	
2014 Existing conditions	Not included	1.60	1.86	3.46
Baseline (*Strategy 2040*)	Not included	2.03	3.05	5.08
Reference (LEAM)	0.02	2.83	2.63	5.48
Mitigation zoning (LEAM)	0.01	2.72	2.46	5.19

## Discussion

### GHG emissions driven by land-use changes and *Strategy 2040* goals

The 2040 GHG emissions estimated by the coupled social–ecological system modeling in the LEAM reference scenario are 7.9% higher than those simply extrapolated from current trends in the *Strategy 2040* baseline scenario (Table 2). The *Strategy 2040* extrapolation is based on official population growth projections and past change trends in per capita emissions, while LEAM forecasts spatially explicit land-use changes to capture the future GHG emissions associated with such changes in urban form. While such changes may be relatively small in Stockholm City and County, they may be considerably larger in other urban regions, with the LEAM-based approach developed and used in this study being important for facilitating assessments and comparisons of such regional conditions.

In the Stockholm case study, the LEAM reference scenario emissions are higher than those in both the *Strategy 2040* baseline scenario and the LEAM MZ scenario for three main reasons, with general relevance. (1) Urban expansion moves into occupying previous natural areas, resulting in loss of carbon sinks. (2) Urban sprawl takes place in suburban areas that traditionally favor single-family residence developments, resulting in higher per capita emissions from the energy use of buildings. (3) Urban sprawl also increases the per capita travel demand, resulting in relatively high per capita VKT and associated transportation GHG emissions.

The fact than GHG emissions are higher in the LEAM reference scenario than those extrapolated in the baseline scenario indicates the simple extrapolation method as non-conservative and rather tending to be insufficient for capturing the full future GHG emissions from urban expansion and associated land-use changes. Such simple extrapolation methods also are not readily useful for testing different policy and management scenarios (especially not spatially explicit ones) and may fail in guiding relevant, efficient measures for emission mitigation.

As an important example of spatially explicit policy instrument, the MZ scenario can specifically target reduction of urban growth in areas with high emission or high carbon sink potential. Although the GHG emission reductions relative to the reference scenario are less than 10% in the Stockholm case (Table 2), this may still be an essential mitigation contribution to the urban and regional spatial planning, extending and complementing the total set of policy instruments and measures required to attain the carbon-neutral goal of *Strategy 2040*.

For other parts of the world, Ewing and Hamidi (2015) present evidence that, e.g., in US cities, 9% of VKT could be reduced if compact growth strategies were adopted instead of urban sprawl. The potential to cut emissions in Stockholm is lower because this and other European cities have historically embraced a less sprawling pattern than US cities. However, spatial growth management policies may still be important for Stockholm to accommodate future population and employment growth while also achieving the region’s and the city’s ambitious climate and carbon neutrality goals.

### Relevance for policy implementation

**Fig. 6:**
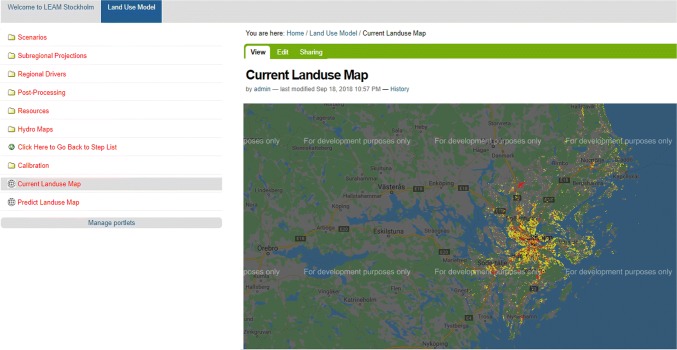
Screenshot of the online LEAM planning support system (PSS) for Stockholm (http://portal.leam.illinois.edu/stockholm2017)

This study was performed together with stakeholders from Stockholm County. An important aspect of our integrated social–ecological modeling approach is the adequacy of information provided to support policymakers, such as the impacts of MZ policy. In an effort to facilitate such communication in the practical policy domain, a planning support system (PSS) was developed to operationalize the modeling work and provide its outcomes for further policy analysis. The PSS interface is available at http://portal.leam.illinois.edu/stockholm2017/ and the scenarios analyzed in this paper are available in Supplementary Materials S1. A screenshot of the PSS interface is shown in Fig. 6.

The development of the online PSS for Stockholm brings two major advantages to further research on climate action planning. First, it can be used to explore and show the consequences of different land-use development scenarios in tangible and objective ways that can challenge habitual ways of thinking and unsustainable development patterns (Deal et al. 2017b). The display of land-use development and assessment of resulting emissions in the PSS can help researchers and policymakers to better understand the costs associated with each development decision. The PSS can also enable “continuous planning” whereby data and models are continuously accessed, examined, and communicated, so that the success/failure pathways of alternative and complementary policies can be determined and re-assessed (Kalantari et al. 2017a, b, 2019; Pan and Deal 2019; Yang et al. 2019). The LEAM–PSS model can readily be updated as new land-use or census data, or new information on planning decisions and follow-up monitoring, become available.

The PSS can also be used to facilitate a mutual learning process on environmental sustainability among cities worldwide (Pan et al. 2018b). Stockholm is one of the pioneers in climate planning, and its deeper understanding of future urban growth and GHG emissions can be applied to other cities by replicating the online cloud-based PSS with a localization process. In order to effectively communicate with local stakeholders via the PSS interface, it is essential to build the model with local stakeholders, involving them in tuning model parameters and validating model results. The information shown to non-expert stakeholders should be easy to understand, such as visual maps of model forecasts of some policy scenarios or some readily understandable quantitative outcomes.

## Conclusions

We present a social–ecological process and systems modeling framework for examining future GHG emissions of various urban development and growth scenarios. The *Strategy 2040* of Stockholm City (2016) sets an overarching goal of net-zero GHG emissions by 2040. Our results show that spatial urban growth and associated changes in land use, carbon sink decreases, and energy uses in buildings and for transportation pose additional challenges to the net-zero emissions goal, unaccounted for in the regional and city development planning. In particular, single-family housing developments associated with urban sprawl have the strongest emission impacts of all forms of urban development. Simulated feedback loops between impacts and policies can improve cause–effect evaluation and support understanding and quantification of alternative policy impacts, such as those of zoning policies that spatially restrict some urban developments. As such, these types of simulations can be an important instrument for GHG emission mitigation. A visualized interface of PSS can also facilitate communication among scientists, policymakers, and other stakeholders, and between cities worldwide willing to take part in mutual learning exercises to improve urban and climate action planning.

Several steps can be taken to extend this research. For example, the GHG emission assessment can be extended by including a human adaptation component to various aspects of climate change (such as flooding, drought, and other extreme events). A comparative modeling framework can improve and extend the current approach by further exploration of modeling assumptions across multiple cities with different development patterns and cultures, such as propensity for sprawl or local preferences for transportation modes.

## Electronic supplementary material

Below is the link to the electronic supplementary material.

Supplementary material 1 (PDF 797 kb)
