# Supplementary material for: Understanding interactions between urban development policies and GHG emissions: A case study in Stockholm Region
Source: Ambio. 2019 Nov 20;49(7):1313–27. doi: 10.1007/s13280-019-01290-y (PMC7190688; doi:10.1007/s13280-019-01290-y)
Supplement: Supplementary file 1 — Supplementary material 1 (PDF 797 kb) [file 13280_2019_1290_MOESM1_ESM.pdf]

Ambio

Electronic Supplementary Material

This supplementary material has not been peer reviewed

Title: Understanding interactions between urban development policies and GHG emissions: A case study in Stockholm Region

Haozhi Pan, Jessica Page, Le Zhang, Cong Cong, Carla Ferreira Elisie Jonsson, Helena Näsström, Georgia Destouni, Brian Deal, Zahra Kalantari*

SUPPLEMENTARY MATERIALS S1

1. Land Use Evolution and Environmental Impact Assessment Model (LEAM)

1.1 Model description

The LEAM model consists of two fundamental organizational parts: (i) a land use change model (LUC) defined by a dynamic set of sub-model drivers that describe the local causality of change, and enable easy addition and removal of variables and the ability to play out ‘what-if’ scenarios; and (ii) impact assessment models that facilitate interpretation and analysis of land use change depending on local interest and applicability, which help to assess ‘so-what’ questions and explore the implications of a scenario. The need in planning and policy making to answer both ‘what-if’ and ‘so-what’ questions is a key basis for the LEAM framework.

In LEAM, the land use transformation potential of individual cells is evaluated by explicitly quantifying the forces (drivers) that contribute to change. Knowledge of the causal mechanisms of change provides local decision makers with the opportunity to test policy and investment choices and is a critical component for completing scenario-planning exercises. Driver submodels are locally dependent and derived through both analysis and local stakeholder interaction. An open architecture and modular design facilitates incorporation of additional local drivers, as needed to improve the explanatory power of the model.

Households and jobs are established and converted into land demand using sector-based economic and demographic analysis, in lieu of sub-regional constraints on demand to determine spatial allocation used in other approaches. The estimated demand serves as a target for regional land allocation. Market variables increase or decrease development rates based on how well the

regional demand targets are met or not met. Simulated outcomes are described in graphs, charts, text, and map form, and are used in engaging local dialogue and in analyzing the potential implications of the changes described. The environmental, economic, and social system impacts of alternative scenarios can be modeled and tested. Scenario descriptions of alternative land use policies, investment decisions, growth trends, and unexpected events (among others) can be simulated, analyzed, and compared for regional importance. LEAM's visual and quantitative representation of each scenario's outcome provides both an intuitive means of understanding and a basis for analyzing the implications of potential decisions. These representations act as a catalyst for discussion and communal decision making.

1.2 Calibration

Calibration is the key to identifying areas for re-development and development. LEAM uses a stochastic greedy algorithm (SGA) to measure connectivity to city and employment centers for current land use cells, and uses the outcome to identify highly probable re-development land areas among existing developed land. The SGA algorithm and our calibration process are introduced below.

1.2.1 Stochastic Greedy Algorithm (SGA) for Accessibility Calculation

Generation of new development probabilities in LEAM includes population centers, employment centers, and other attractions (can be self-defined by users) as attractants or repellents to new developments. The original data types for those attractions are point data, although in LEAM their influence is not constrained as points. For example, people may prefer to live close to employment centers for shorter commuting time and better job opportunities. Thus, it is

necessary to include spatial dependencies of accessibility to important attractions in the considerations.

The conventional spatial matrix uses direct neighborhood or straight-line distance as the cutoff. However, in our case, it is more appropriate to use the shortest travel time from each cell to each attractor as a proxy for spatial weight. The typical method to calculate shortest travel times uses the Dijkstra gravity algorithm (Goodman et al. 2016). In this case, our densely connected raster of *n* cells (more than 1 million cells in the Stockholm region) means a computational complexity of $O(n^2)$ for each attractor (Goodman et al. 2016). This suggests that the calculation time of the Dijkstra algorithm increases by a power of two for total number of cells, which means that the calculation would be computationally expensive and take a long time for each of the (10 or so) attractors used in our model.

An alternative to Dijkstra is the greedy algorithm approach. A greedy algorithm has a much smaller computational complexity of $O(n)$ for the same shortest travel time calculation. A greedy algorithm sends agents out from a given attractor in random patterns until it discerns the most efficient pathway. The approach finds local optima instead of Dijkstra's more overall optima, which means that the algorithm sacrifices performance for computational efficiency. To maintain the balance between performance and efficiency, LEAM uses a parallel SGA to determine attractor travel times. Basically, hundreds of greedy algorithms are run simultaneously. Each is assigned a randomized decision rule and a chance to "jump" out of its local optimization routine to find a more globalized optimization (Viswanathan et al., 2011). **Figure S1** presents summary logic of the SGA process for finding the shortest (TT) path from one population center *k* to other cells.

```
Algorithm SGA{
    Initiate every cell on the map with infinity travel time;

    Repeat the following N times{
        Initiate a direction;

        Repeat the following T steps{
            Agent from current cell moves to an adjacent cell, with higher
            probability to the cell with lesser travel barrier and in accordance
            with the original direction #agent from population center k in the first
            step
        }
    }
    Update the least travel time from population center k for every cell on the map
}
```

Figure S1. Summary logic of a stochastic greedy algorithm (SGA) approach for shortest travel time calculations.

As noted, the computational complexity for the SGA algorithm is *O(TN)*, but in this case *N* is the number of iterations a modeler specifies greedy algorithms to take and *T* is the distance cutoff that is applied (since one cell is 30x30m, the modeler can specify that locations more than 1,000 steps (30,000 m) away from a population center do not matter). Note that *N* and *T* are significantly smaller than the total number of cells, *n*, and thus SGA has a much smaller computational cost than Dijkstra. Further, *N* processes of randomized agent dispatching have no dependencies, so it is possible to parallel the N processes, reducing the computational time to *O(TN/C)* (where C is the number of threads available for parallelization). Several attractors can be run simultaneously, which can potentially run almost as fast as one greedy algorithm. An example of an SGA process with pseudo-coding is shown in **Figure S2**.

Algorithm SGA{

Initiate every cell on the map with infinity travel time;

Repeat the following 1,000 times{

Randomly draw a direction ***d*** *from direction set* ***M****={N, NE, E, SE, S, SW, W, NW} with equal chance; # N—North, E—East, S—South, W---West, NE—Northeast, etc;*

Repeat the following 1,000 steps{

Define ***P*** *as a probability vector with {*$\boldsymbol{p}_N$, $\boldsymbol{p}_{NE}$, $\boldsymbol{p}_E$, $\boldsymbol{p}_{SE}$, $\boldsymbol{p}_S$, $\boldsymbol{p}_{SW}$, $\boldsymbol{p}_W$, $\boldsymbol{p}_{NW}$*}, where every element of* ***P*** *is a continuous number in [0, 1];*

Create set of neighboring direction vectors of d as ***E****={*$\boldsymbol{e}_1$, $\boldsymbol{e}_2$, $\boldsymbol{e}_3$*}; # for example, if* ***d*** *is NE, then* ***E****={N, E, NE}*

Randomly draw two directions from ***E*** *as vector* ***D****={*$\boldsymbol{d}_1$, $\boldsymbol{d}_2$*} with equal probability; #in this case, assume NE is the direction* ***d*** *and D={****NE, N****}*

Assign the probability P for a cell to move in the direction of each of the elements of D as 0.35; for the two directions adjacent to E, but not in E, as 0.1; for the remaining four directions as 0.025; In our example, ***P****={*$\boldsymbol{p}_N$, $\boldsymbol{p}_{NE}$, $\boldsymbol{p}_E$, $\boldsymbol{p}_{SE}$, $\boldsymbol{p}_S$, $\boldsymbol{p}_{SW}$, $\boldsymbol{p}_W$, $\boldsymbol{p}_{NW}$*}={0.35, 0.35, 0.025, 0.09, 0.025, 0.025, 0.025, 0.09};*

Assign a ***L*** *probability vector to each direction based on the travel barrier of the land use type on each cell;*

Calculate the final direction moving probability ***Q****={*$\boldsymbol{q}_i$*:* $i \in N$, $q_i = p_i l_i / \sum_{j \in N} p_j l_j$*};*

Agent from current cell moves to an adjacent cell with probability vector ***Q*** *to {N, NE, E, SE, S, SW, W, NW};*

}

}

Update the least travel time from population center k for every cell on the map

}

Figure S2. More detailed stochastic greedy algorithm (SGA) logic for shortest travel time calculations.

1.2.2 Calibration of Existing Land Use to Accessibility

To consider the size of population/employment centers for accessibility, we use **equation 1** to convert travel time into an attractiveness score.

$$a_{ik} = \frac{p_k}{c_{ik} + l} \quad (1)$$

where a_{ik} is the attractiveness score from center k for cell i; p_k is the total population of center k; c_{ik} is the travel time **cost** from cell i to center k; and l is a normalization factor. In this case, l is set to be 20 minutes, which means travel time from a center of less than 20 minutes does not make a major difference in terms of attractiveness from the center for the cell.

It is clear that employment accessibility is concentrated in the center of the city of Stockholm, while population centers are more disperse. We hypothesize that the actual land use distribution might also follow this trend. To test this, we plot the occurrence of commercial and residential land uses in Stockholm against the travel time to employment and population centers, as well as transportation, water, and forest attractors (see **Figures S3-S7)**.

Probability of residential cells vs employment attrator values

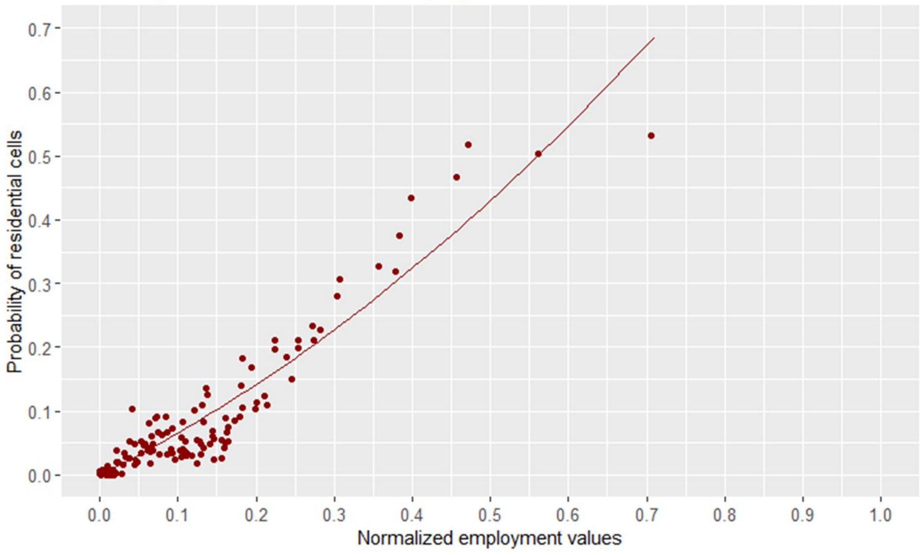

Figure S3. Frequency of existing residential cells to employment centers by attraction values.

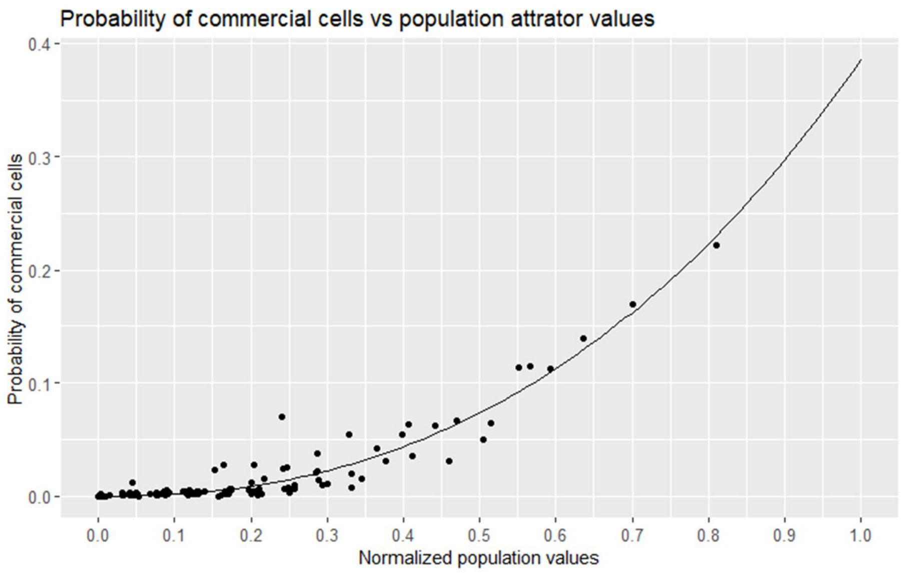

Figure S4. Frequency of existing commercial cells to population centers by attraction values.

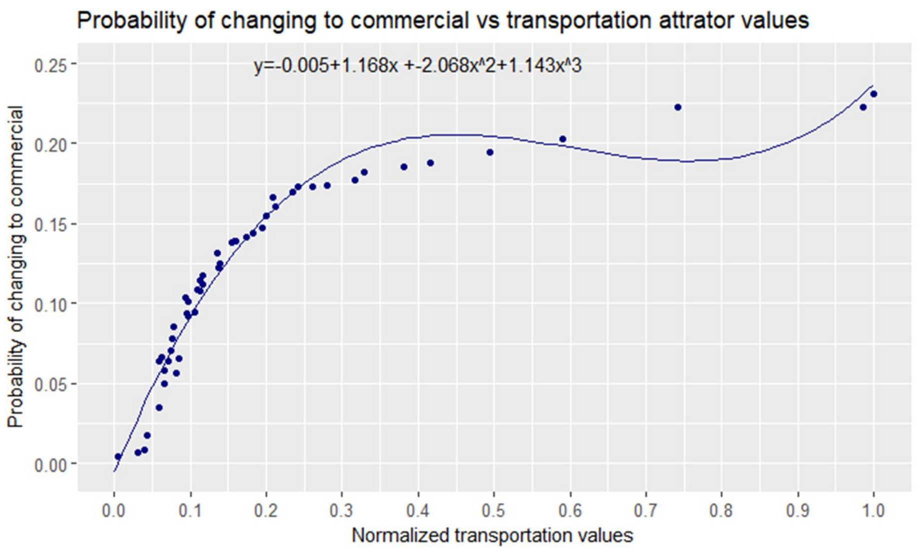

Figure S5. Frequency of existing commercial cells to transportation centers by attraction values.

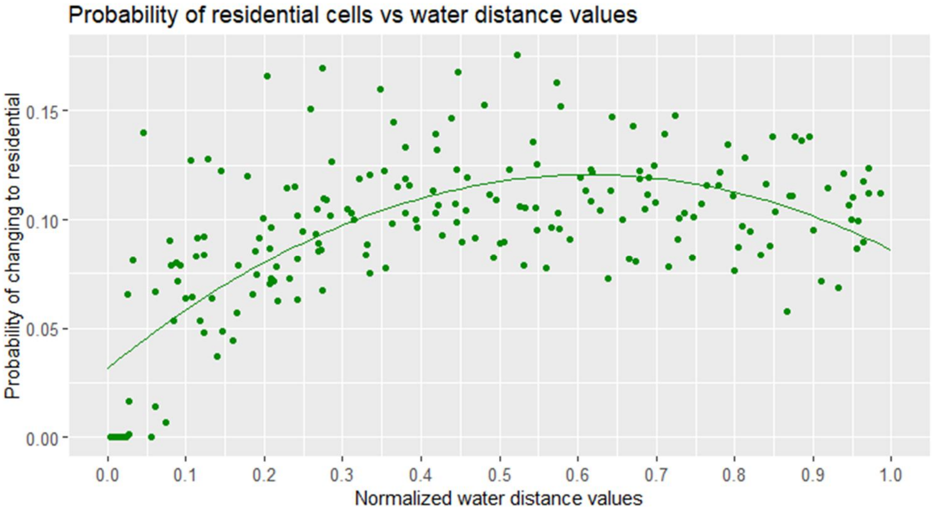

Figure S6. Frequency of existing residential cells to water by attraction values.

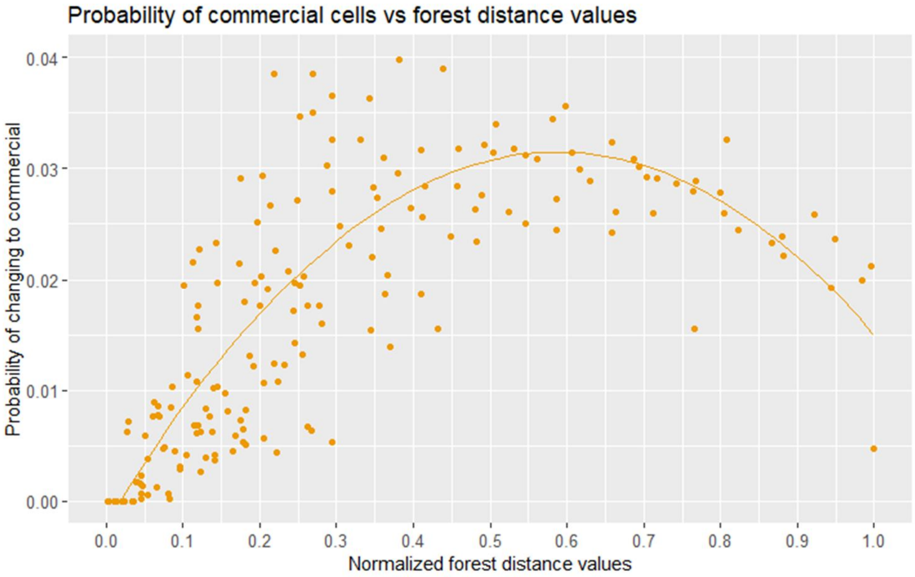

Figure S7. Frequency of existing commercial cells to forests by attraction values.

Building Probability Maps

Using the relations calibrated above, we build a new probability map for residential and commercial development for Stockholm. Residential and commercial developments are allocated based on the highest probability as well as the demand.

3. Validation

For validation, variable weighting, and calibration, we use a modified multi-resolution fitting process. Additionally, Deal et al. (2017) argue that PSS-based scenario planning processes and outcomes can be improved by including the ability to do multi-directional temporal analyses, such as “re-casting” from a point in time in the past to the current condition (Deal et al., 2017). By re-casting, modelers can compare past predictions with current “ground-truth” conditions. This comparison requires a reliable validation method. It enables modelers to pinpoint parts of the model that need improvement, to weigh variables correctly, and to support and validate future forecasts. For planners, it can also validate (or invalidate) assumptions made regarding past policies.

We use a modified multi-resolution fitting process to communicate model goodness-of-fit. Model evaluations are performed using an expanding window to gradually degrade the resolution of the comparison, yielding information not contained in single resolution methods. The method can provide a clearer picture of how a model performs in each smaller, specific spatial zone, without losing information as in conventional cell-by-cell level statistical approaches. We use zonal constructs that are based on statistical patterns, using general goodness-of-fit criteria and applied to different (multiple) scaled zones simultaneously in order to calibrate and validate modeled outcomes. The approach allows goodness-of-fit to be examined at different spatial

resolutions and mitigates the arbitrariness of both visual comparisons and spatial boundary selection in a way that is both objective and reasonable.

2. Carbon Sink Mapping of Stockholm

We produce a carbon sink map of the region based on the latest available land use map (with data from TRF and the Swedish Land Survey Authority (*Lantmäteriet*), considering six classes: forest, shrubs, grass, cultivated crops, pasture, and wetlands (including both woody wetlands and herbaceous wetlands). Within forest areas, carbon sink values are also assigned considering different vegetation types and ages, given the carbon sequestration potential of (i) young and productive forests, and (ii) established or naturally occurring forests. Although there is a considerable amount of productive forest in Sweden, only 0.3 percent of this is young productive forest. Thus, carbon sequestration potential of the average age of established, non-productive forest is assumed.

In addition to the data provided by TRF, we produce a carbon sink map of the region based on the latest available land use map (updated at Stockholm University with data from TRF and Lantmätariet[1]), with carbon sink values assigned to each different vegetation type and land use identified in the map, as shown in **Figure S8**.

[1]From https://www.lantmateriet.se/sv/Kartor-och-geografisk-information/

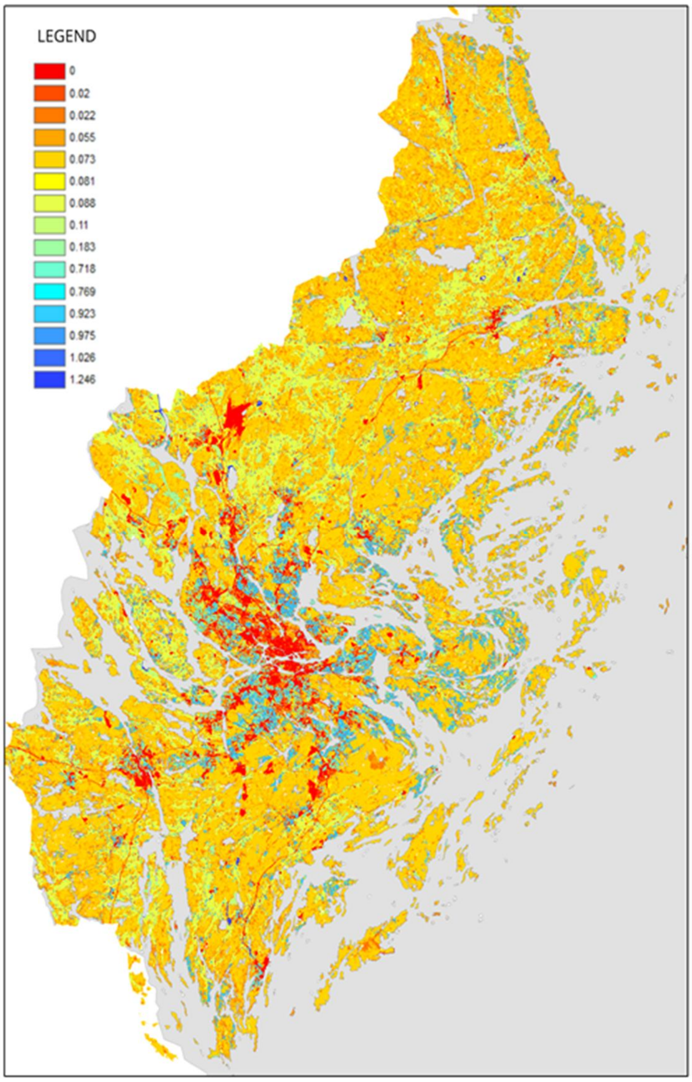

Figure S8. Map showing the carbon sink potential (kgCO_2eq/m^2/year) of the land in Stockholm County. The grey areas are water.

3. Detailed Methods for Estimating GHG Emissions

3.1 Building Emissions

For each land use cell simulated to develop into new urban built-up area (from 2010 to 2040) in Stockholm, the carbon implications from its estimated end-use are evaluated. *Strategy 2040* is

used for projected carbon emissions and energy use from each building type (e.g., single family houses, multi-family condos, or apartments), by assigning density zones (extracted from RUFS 2050) to newly developed residential and commercial buildings. Based on this new spatial information, building emissions are assessed for each land use cell and summed to a new total. The updated building emissions forecast is then compared against that in the *Strategy 2040* baseline scenario.

One assumption in *Strategy 2040* and other future GHG emissions assessments is that newly built residential buildings are proportional to population growth. The only change in the trend of residential GHG emissions comes from implementation of new building energy standards. In *Strategy 2040*, annual energy consumption per square meter drops from 110-160 GWh for existing buildings to 42 GWh for new buildings. This assumption is tested here by re-assessing development type and housing area per person based on the spatial location of simulated future residential development. New residential developments are overlaid on the density map of Stockholm and 1.5 persons per cell (equivalent to 1667 people per square kilometer) is used as the threshold for whether the new cell hosts multi-family houses (higher density development) or single-family houses (lower density development). For single-family houses, the building technology assumption is taken from the *Strategy 2040* (i.e., annual energy consumption of 42 GWh/m^2). For multi-family homes, building utility intensity can be expected to be much higher and thus the model assumes that energy consumption for multi-family homes is equal to 60 GWh per square meter and year. It is noteworthy that, despite higher energy use per square meter, multi-family buildings still have a favorable trade-off in terms of GHG emissions impacts, because they have a much higher density of residents and decrease the total demand per resident for new buildings.

3.2 Transportation Emissions

In the present study, we adapt the Hankey and Marshall (2010) method to model links between the LEAM forecast for future land use pattern and GHG emissions in Stockholm. First, we recalibrate the Hankey and Marshall (2010) linear population density functional relationship between VKT and urban form, using existing population density in Stockholm and current VKT from *Strategy 2040*. The same functional relationship is extrapolated to the 2040 LEAM land use scenario. The impacts of the future public transport network are then evaluated. The model estimates two options: (1) commuting needs to urban growth on the outskirts of Stockholm are satisfied solely by passenger cars; and (2) the public transportation network is expanded to newly developed areas with the same usage rate as the existing conditions. The case of a public transportation network is used in the reference scenario, because it is a more likely future based on the climate commitments of the City of Stockholm. Modeling results regarding GHG emissions provided by both residential/commercial buildings and transportation for the 2040 reference scenario are compared with the baseline scenario in *Strategy 2040*.

4. Policy Scenario and Feedback Modelling

The policy instrument designated in this model is special policy zones that restrict residential and commercial developments. Several types of special zones are identified and simulated by LEAM. The first zone type is future flood zones under expected climate change scenarios. This type of no-growth zone simulates policymakers' awareness of climate change impacts and prompts them to actively adapt future development strategy for climate mitigation. In the second type, the 2040 flooding zone is simulated by a hydrological model (r.sim.water) and a hydrodynamic model (MIKE FLOOD) with LEAM land use change inputs. This type of no-growth zone simulates

policy-makers' awareness of the necessity for climate mitigation. Areas associated with high emissions potential (such as large patches of forests occupied by urban development, regions far from urban cores, or single-family residence areas) are then set as no-growth zones. As a result, residential and commercial developments are shifted to places with lower emissions potential, although socio-economic attractiveness (measured by the LEAM probability map) is slightly lower in those areas. The third type of no-growth zone is called other no-growth zones, and includes forest preserves, parks, and water bodies.

In the 2040 reference scenario, land use change has a one-way effect on carbon emissions and climate change, and thus no feedback loop is considered. In the mitigation zoning (MZ) scenario, land use change probabilities are affected by potential climate impacts through restrictive zoning policies. The feedback effects further update the land use simulation and have profound impacts on GHG in future time-steps of the simulation. In this way, a full loop between land use, climate change, and policy reactions is constructed to update the resulting GHG emissions impacts. Model results on GHG emissions in the MZ scenario for 2040 are compared with current best-practice climate planning on municipal scale using the *Strategy 2040*. The major improvement of the MZ scenario compared with the reference scenario of the current *Strategy 2040* is that the urban growth management strategy in the MZ scenario uses the information from model results. The final MZ scenario result after iterations then indicates the emissions savings that can be achieved by applying the zoning strategy.

References

Deal, B., Pan, H., Timm, S., & Pallathucheril, V. (2017). The role of multidirectional temporal analysis in scenario planning exercises and Planning Support Systems. *Computers,*

Environment and Urban Systems, 64, 91–102. doi: doi.org/10.1016/j.compenvurbsys.2017.01.004

Goodman, L., Lauschke, A., & Weisstein, E. W. (2016). Dijkstra's Algorithm. MathWorld--A Wolfram Web Resource. Retrieved at http://mathworld.wolfram.com/DijkstrasAlgorithm.html

Hankey, S., and J. D. Marshall. 2010. Impacts of urban form on future US passenger-vehicle greenhouse gas emissions. Energy Policy 38: 4880–4887.

Viswanathan, V., Sen, A. K., & Chakraborty, S. (2011). Stochastic Greedy Algorithms. *International Journal on Advances in Software Volume 4, Number 1 & 2, 2011*. doi: 10.1.1.675.4270
